# The effect of interleukin-22 treatment on autoimmune diabetes in the NOD mouse

**DOI:** 10.1007/s00125-017-4392-2

**Published:** 2017-08-04

**Authors:** Danielle J. Borg, Ran Wang, Lydia Murray, Hui Tong, Raymond J. Steptoe, Michael A. McGuckin, Sumaira Z. Hasnain

**Affiliations:** 1 Inflammatory Diseases Biology and Therapeutics, Mater Research Institute – The University of Queensland, Translational Research Institute, Level 4/37 Kent Street, Woolloongabba, Brisbane, QLD 4102 Australia; 2 Tolerance and Autoimmunity Group, University of Queensland Diamantina Institute, Translational Research Institute, Brisbane, QLD Australia

**Keywords:** Autoimmune diabetes, IL-22, NOD mouse

## Abstract

**Aims/hypothesis:**

The aim of this study was to determine whether therapy with the cytokine IL-22 could be used to prevent the development of, or treat, autoimmune diabetes in the NOD mouse.

**Methods:**

Six-week-old NOD mice were administered bi-weekly either recombinant mouse IL-22 (200 ng/g) or PBS (vehicle control) intraperitoneally until overt diabetes was diagnosed as two consecutive measurements of non-fasting blood glucose ≥ 11 mmol/l. At this time, NOD mice in the control arm were treated with LinBit insulin pellets and randomised to bi-weekly therapeutic injections of either PBS or IL-22 (200 ng/g) and followed until overt diabetes was diagnosed, as defined above.

**Results:**

IL-22 therapy did not delay the onset of diabetes in comparison with the vehicle-treated mice. We did not observe an improvement in islet area, glycaemic control, beta cell residual function, endoplasmic reticulum stress, insulitis or macrophage and neutrophil infiltration as determined by non-fasting blood glucose, C-peptide and histological scoring. Therapeutic administration of IL-22 did not reduce circulating lipopolysaccharide, a marker of impaired gut mucosal integrity.

**Conclusions/interpretation:**

Our study suggests that, at this dosing regimen introduced either prior to overt diabetes or at diagnosis of diabetes, recombinant mouse IL-22 therapy cannot prevent autoimmune diabetes, or prolong the honeymoon period in the NOD mouse.

**Electronic supplementary material:**

The online version of this article (doi:10.1007/s00125-017-4392-2) contains peer-reviewed but unedited supplementary material, which is available to authorised users.

## Introduction

In an attempt to prevent or reverse type 1 diabetes, basic and clinical research has focused intensely on immunotherapies, beta cell replacement and preservation of beta cell function and mass [1]. The complexity of type 1 diabetes pathogenesis [2] and the human beta cell [3] has shifted the focus to better understanding disease heterogeneity. Most recently, post-translational modifications of beta cell proteins have been identified as ‘neo-antigens’ in the NOD mouse [4] and individuals with type 1 diabetes (reviewed in [5]), further contributing to the complexity of this disorder.

The mechanism of why and how neo-antigens are produced remain unknown. It has been hypothesised that the normal physiological role of beta cells as highly secretory cells, when combined with various environmental stimuli, significantly increases oxidative and endoplasmic reticulum (ER) stress. This leads to MHC presentation of secretory pathway proteins and neo-antigens, precipitating disease in individuals with a genetic predisposition to autoimmunity [5]. Thus, therapies with the ability to reduce beta cell oxidative stress, protein misfolding and ER stress may limit neo-antigen production, reducing the likelihood of activation of potentially pathogenic neo-antigen-specific T cells that are subject to normal tolerance mechanisms [5]. Furthermore, once autoimmunity develops, beta cell damage is largely driven by T cell cytokines that drive oxidative stress and consequent beta cell apoptosis, providing another potential benefit of therapies inhibiting oxidative stress.

We previously demonstrated that the cytokine IL-22 directly reduces pancreatic beta cell oxidative and ER stress caused by cytokines and glucolipotoxicity in murine and human islets that highly express the IL-22 receptor [6]. When IL-22 therapy was administered in the short term to obese hyperglycaemic mice, we and others observed a reduction in hyperglycaemia [6, 7], which was associated with suppression of islet ER stress and inflammation, promotion of high-quality insulin and restoration of glucose homeostasis [6]. To address whether IL-22 therapy could reduce beta cell stress and prevent the development of autoimmune diabetes, or treat established disease, we used the NOD mouse experimental model of type 1 diabetes. We investigated whether, using this dosing regimen, prophylactic IL-22 therapy would prevent diabetes, and whether therapeutic IL-22 administration would improve beta cell function or gut integrity in the NOD mouse.

## Methods

### Experimental animals

All procedures were performed in accordance with guidelines from the National Health and Medical Research Council of Australia and the University of Queensland (UQ). Experimental protocols involving mice were approved by the UQ Animal Ethics Committee (TRI/MRI-UQ/333/14/NHMRC/MF). Four-week-old female NOD mice were purchased from the Animal Resource Centre (Perth, WA, Australia) and given ad libitum access to standard chow and water. At 6 weeks of age, the mice were randomised (to ensure the presence of treatment and control animals in the same cages) into groups receiving either 200 ng/g i.p. recombinant mouse IL-22 (rmIL-22; Cell Signaling, Danvers, MA, USA) or an equivalent volume of PBS bi-weekly.

A subset of mice (*n* = 4) were euthanised 30 min after rmIL-22 administration to assess activation of signal transducer and activator of transcription 3 (STAT3) phosphorylation in the pancreas. Body weight and non-fasted blood glucose concentration were monitored between 09:00 and 11:00 h twice weekly.

From day 100 of life, the mice were monitored daily for diabetes. Diabetes was confirmed after two consecutive measurements of ≥ 11 mmol/l glucose from non-fasting blood samples from the tail. At the confirmation of diabetes, rmIL-22-treated mice were euthanised, and whole blood, for serum isolation, and pancreas were collected. Upon confirmation of diabetes in the PBS control-treated group, mice were treated with a one-and-a-quarter dose of LinBit insulin pellets according to the manufacturer’s instructions (one dose is approximately 0.1 U insulin/24 h; LinShin Canada, ONT, Canada) and randomised to receive rmIL-22 or PBS twice weekly as in the prophylactic experiment. LinBit insulin pellets were replaced on two consecutive non-fasting blood glucose measurements of ≥ 11 mmol/l, up to a total of three times. Upon expenditure of the third insulin pellet, after two consecutive non-fasting blood glucose measurements of ≥ 11 mmol/l or at the end of the study (day 265 of life), mice were euthanised and tissues taken as described above. The analyses were conducted without knowledge of group assignment and outcome assessment.

### Bioassays

Glucose was measured from spot blood taken from the tail of the mice using a glucometer (SensoCard; Point of Care Diagnostics, Artarmon, NSW, Australia). Serum at the time of diabetes diagnosis was used to measure C-peptide (Crystal Chem, Downers Grove, IL, USA) and, after heat inactivation, lipopolysaccharide (LPS; Lonza, Mt Waverley, VIC, Australia) according to the manufacturers’ instructions.

### Histology

Frozen pancreases were sectioned (4–5 μm thickness; HM525NX Cryostat, Thermo Fisher Scientific, Scoresby, VIC, Australia), fixed with 100% ethanol at room temperature for 10 min and allowed to dry for 15 min at room temperature. Sections were stained using H&E, imaged (×40 dry objective, VS120 Virtual Slide Microscope; Olympus Australia, Notting Hill, VIC, Australia) and quantified for insulitis and insulitis index [8] (OlyVIA software version 3.7, Olympus Australia). A range of 22–37 islets, spaced over 25 μm apart, over four serial sections were imaged per mouse. Standard immunofluorescence staining methods previously described [9] were used to assess the levels of ER stress (glucose-related protein 78 [GRP78] antibody; sc-1050; Santa Cruz Biotechnology, TX, USA; 1:25), macrophages (F4/80 antibody; CI:A3-1; Abcam, Cambridge, UK; 1:200), neutrophil infiltration (Ly6G6c antibody; NIMP-R147, Abcam; 1:200) and STAT3 phosphorylation (STAT3p antibody; D3A7, Cell Signaling, Danvers, MA, USA; 1:500). The area in pixels/mm^2^ was quantified using 3–4 islets per mouse using Image J software version 1.45 s.

### Statistical analyses

In the prophylactic experiment, time to diabetes data were analysed using survival methods and logrank tests. In the therapeutic experiment, delay to diabetes data were analysed using logrank tests and the Mann–Whitney *U* test for non-parametric data. Proportions of histological insulitis scores were analysed using a *χ* ^2^ test with a Bonferroni test for multiple comparisons. Non-parametric biochemical data were analysed using a Mann–Whitney *U* test. Linear regression and Spearman’s rank order test for correlation were used to analyse relationships with biochemical data over time or between biochemical analyses, respectively. No data were excluded from the analysis. Data are presented as either means ± SD, or as box plots where median (line), first and third quartile distributions (box), and minimum and maximum values (whiskers) and individual mice are shown. A *p* value <0.05 was considered significant.

## Results

**Fig. 1:**
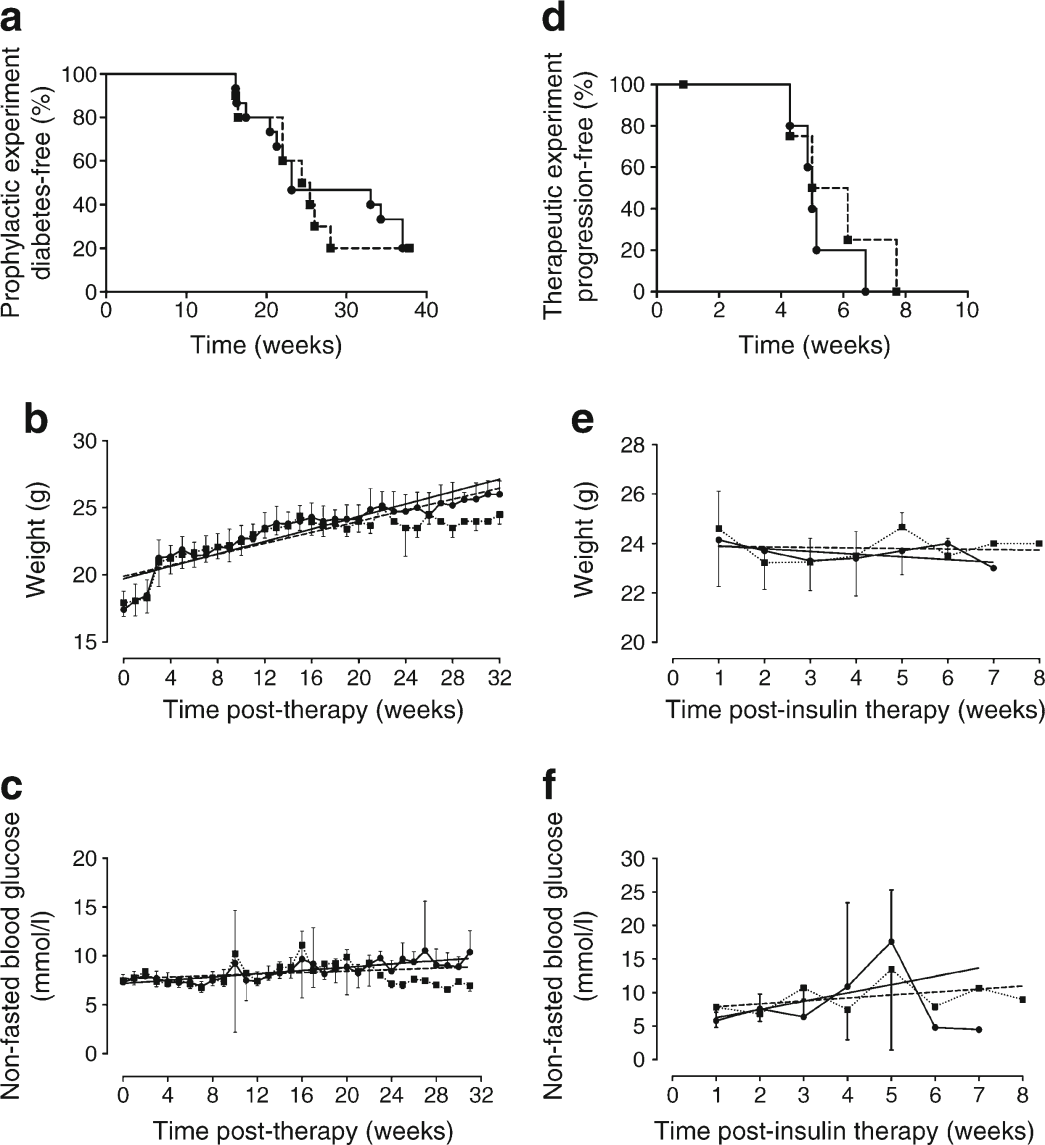
(**a**) Time to diabetes incidence *(p* = 0.82), (**b**) body weight, and (**c**) non-fasting blood glucose of NOD mice treated from 6 weeks of age bi-weekly with either vehicle (PBS, black circles, solid line *n* = 15) or 200 ng/g rmIL-22 (black squares, dashed line *n* = 10). (**b**) *p*_slope_ = 0.10, *p*_elevation_ = 0.35. (**c**) *p*_slope_ = 0.06, *p*_elevation_ = 0.99. (**d**) Time to diabetes progression (*p* = 0.54), (**e**) body weight, and (**f**) non-fasting blood glucose of NOD mice therapeutically treated with insulin and with IL-22 as described above from the time of first diabetes diagnosis (*n* = 4–6 per group). (**e**) *p*_slope_ = 0.65, *p*_elevation_ = 0.59. (**f**) *p*_slope_ = 0.43, *p*_elevation_ = 0.90. Data are means ± SD. Solid (PBS) or dashed (rmIL-22) lines represent linear regression. Statistical tests: logrank test, linear correlation

Prophylactic therapy with rmIL-22 for 32 weeks failed to delay or prevent the development of overt diabetes in NOD mice (rmIL-22, 8 out of 10; PBS, 12 out of 15; *p* = 0.82, logrank test; Fig. 1a). Median diabetes-free survival in mice treated with rmIL-22 was 175 days (95% CI 115, 265 days) compared with 162 days (95% CI 143, 265 days) in the vehicle control-treated mice (*p* = 0.90, Mann–Whitney *U* test; Fig. 1a). rmIL-22 therapy for 32 weeks did not result in an alteration in body weight (*p*_slope_ = 0.095, linear regression; Fig. 1b) or non-fasted blood glucose (*p*_slope_ = 0.057, linear regression; Fig. 1c), compared with vehicle-treated mice.

After overt diabetes had been confirmed in animals treated in the prophylactic control arm, mice were given suboptimal insulin therapy (LinBit insulin pellets) and reallocated to receive therapeutic doses of either rmIL-22 or vehicle to determine whether therapy could prolong the ‘honeymoon phase’ of diabetes progression. At the time of randomisation, the mice did not significantly differ in age (PBS, 173 days, 95% CI 110, 235 days; rmIL-22, 166 days, 95% CI 98, 235 days). In the therapeutic intervention arm, rmIL-22 failed to significantly prolong median time to diabetes progression (39 days, 95% CI 6, 54 days; *n* = 5; Fig. 1d) in comparison with the vehicle-treated animals (35 days, 95% CI 6, 47 days; *n* = 6; *p* = 0.46, logrank test; Fig. 1d). Throughout this therapeutic phase of the study, rmIL-22 intervention did not significantly affect body weight (*p*_slope_ = 0.65, linear regression; Fig. 1e) or non-fasted blood glucose (*p*_slope_ = 0.43, linear regression; Fig. 1f) compared with the animals that were randomised to PBS treatment.

**Fig. 2:**
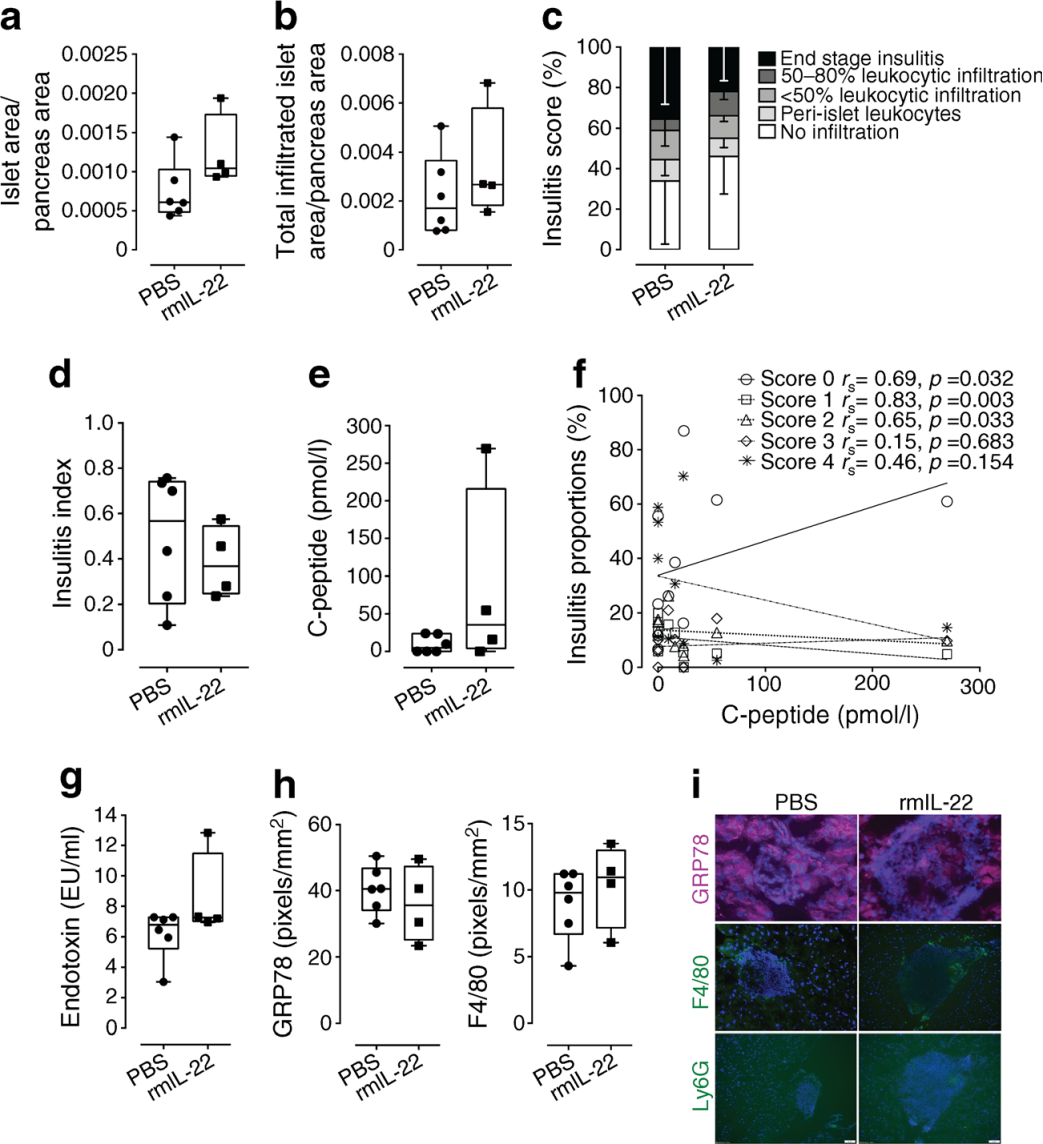
Endpoint analysis of diabetic NOD mice that were treated therapeutically bi-weekly with PBS (circles) or rmIL-22 (squares; 200 ng/g). (**a**) Islet area per pancreas per mouse (*n* = 4 serial sections; *n* = 4–6 per group), *p* = 0.07. (**b**) Islet area including infiltrate per pancreas per mouse; *p* = 0.35. (**c**) Degree of islet infiltrate (*n* = 22 ± 8 to 37 ± 14 islets analysed per mouse per group); *p* for *χ* ^2^ test <0.0001, *p*_interaction_ = NS, *p*_treatment_ = NS; *p*_insulitis_ = 0.0006. (**d**) Insulitis index (0, no immune infiltrate in any islets, 1, end-stage insulitis for all islets), *p* = 0. 70. (**e**) Concentration of circulating fasting C-peptide (*n* = 4–6/group), *p* = 0.27. (**f**) Correlation between insulitis proportions and C-peptide (*n* = 10; treatment groups combined, Spearman’s rank order correlation test). (**g**) Circulating levels of LPS/endotoxin (*n* = 4–6 per group), *p* = 0.15; EU, endotoxin units. (**h**) Quantification of immunofluorescence staining in islets (**i**) to assess the changes in ER stress (GRP78), macrophage and neutrophil infiltration (F4/80 and Ly6G, respectively); scale bars, 50 μm. Box plots represent median, interquartile range and minimum and maximum values. Mean ± SD are shown in (**b**). Statistical tests: Mann–Whitney *U* test, *χ* ^2^ test linear correlation, Spearman’s rank order test for correlation

To determine whether therapeutic administration of rmIL-22 induced changes in the pancreas, pancreases were collected at the time when diabetes progression was confirmed in the therapeutic experiment. At the time of diabetes progression and hyperglycaemia, islet area and total islet area including infiltrate did not increase upon rmIL-22 treatment (*p* = 0.07 and *p* = 0.35, respectively, Mann–Whitney *U* test; Fig. 2a, b). While varying degrees of immune cell infiltrate were observed in islets from both treatment groups (*p* < 0.0001, *χ* ^2^-test; Fig. 2c), rmIL-22 therapy accounted for less than 0.1% of the total variance in insulitis grading (Bonferroni’s multiple comparisons test; Fig. 2c).

No significant differences in the insulitis index were observed (*p* = 0.69; Fig. 2d). Residual beta cell function, as determined by circulating C-peptide, reflected a similar variation, but no significant improvement, after therapeutic administration of rmIL-22 in comparison with vehicle-treated mice (*p* = 0.27; Fig. 2e). Despite the variation observed with fasting C-peptide levels, no correlation was observed with islet area or insulitis index (data not shown). However, C-peptide was positively correlated with higher proportions of non-infiltrated islets (*p* = 0.03, Spearman’s rank order correlation; Fig. 2f) and negatively correlated with lower proportions of peri-insulitis and 50% or less insulitis (*p* = 0.003 and 0.033, respectively, Spearman’s rank order correlation; Fig. 2f). Therapeutic administration of rmIL-22 did not affect intestinal mucosal barrier function as determined by circulating LPS, a surrogate marker for impaired mucosal integrity (*p* = 0.15; Fig. 2g). LPS levels did not correlate with circulating C-peptide or islet pathology (data not shown). No differences were observed in macrophage or neutrophil infiltration, and beta cell staining with the ER stress marker GRP78 was not altered by rmIL-22 treatment (Fig. 2h, i).

## Discussion

Here we used rmIL-22 therapy to determine whether diabetes onset could be delayed or whether hyperglycaemia could be controlled in the NOD mouse. In this small study, we found that neither prophylactic treatment starting at 6 weeks of age, nor therapeutic intervention of rmIL-22 once overt diabetes was confirmed, delayed the onset of diabetes or improved glycaemic control in comparison with vehicle-treated mice.

In contrast to previous findings from our group [6] and others [7] in mouse models of obesity and type 2 diabetes, we observed little change in beta cell function after IL-22 therapy in the NOD mouse. The amount of IL-22 required to overcome beta cell ER stress to limit self-antigen presentation and beta cell loss in autoimmune diabetes may differ substantially from that required to contend with the low-level chronic inflammation in obesity and type 2 diabetes. Greater IL-22 exposure could be achieved by more frequent administration or the use of more metabolically stable hybrid proteins, such as Fc-based fusion proteins, which have been shown to extend the half-life of IL-22 by approximately 3 days [7]. Previous studies demonstrated that deletion of IL-22 suppressed diabetes progression led by autoreactive T cells [10], and that rmIL-22 treatment did not block streptozotocin-induced type 1 diabetes [11]. We confirmed that IL-22 was active and reached the pancreas by assessing downstream STAT3 activation in whole pancreas of NOD mice, 30 min after the administration of 200 ng/g rmIL-22 (see electronic supplementary material [ESM] Fig. 1a). It is also possible that, by 6 weeks of age, when treatment was commenced, NOD mice had been exposed to environmental cues and beta cell ER stress that had already initiated presentation of self-antigens and precipitated T cell-driven autoimmunity.

Of note, IL-22RA1 expression in the pancreas of NOD mice also diminished with ageing (ESM Fig. 1b), which could be due to increased infiltration within the islets. Using a tolerogenic vaccination approach, in utero delivery of a proinsulin Fc fusion protein in G9C8 developing embryos has recently been demonstrated to be beneficial in preventing disease onset [12]. This early delivery method may overcome the issue of treatment timing in this autoimmune model [12] and the barrier of IL-22 placental transport [13]. However, the viability for clinical translation of such early intervention approaches is doubtful.

Circulating LPS remained unchanged after therapeutic delivery of IL-22, in contrast to our previous findings in obese mice, in which the improvement of mucosal integrity by IL-22 therapy led to changes in gut microbiota, improved intestinal barrier function and lowered serum LPS [14]. Leaky intestinal mucosal barrier [15], altered microbiota and reductions in short-chain fatty acids are a common occurrence in both types of diabetes [16, 17]. This lack of change may illustrate disparities in endotoxin/LPS levels in different preclinical rodent models of diabetes, or differences in the detection method used for mucosal integrity. We believe future work should focus on understanding whether different IL-22 dosing regimens or IL-22 fusion proteins would (1) resolve autoimmunity-induced ER stress in the beta cell, and (2) affect microbiota or pancreatic antimicrobial peptides [18], to improve efficacy in autoimmune diabetes.

## Electronic supplementary material

ESM Fig. 1 (PDF 1101 kb)

## Abbreviations

ER: Endoplasmic reticulum
GRP78: Glucose-related protein 78
LPS: Lipopolysaccharide
rmIL-22: Recombinant mouse IL-22
STAT3: Signal transducer and activator of transcription 3
UQ: University of Queensland
