# Supplementary material for: The effect of interleukin-22 treatment on autoimmune diabetes in the NOD mouse
Source: Diabetologia. 2017 Aug 4;60(11):2256–61. doi: 10.1007/s00125-017-4392-2 (PMC6448904; doi:10.1007/s00125-017-4392-2)
Supplement: Supplementary file 1 — (PDF 1101 kb) [file 125_2017_4392_MOESM1_ESM.pdf]

ESM Fig. 1

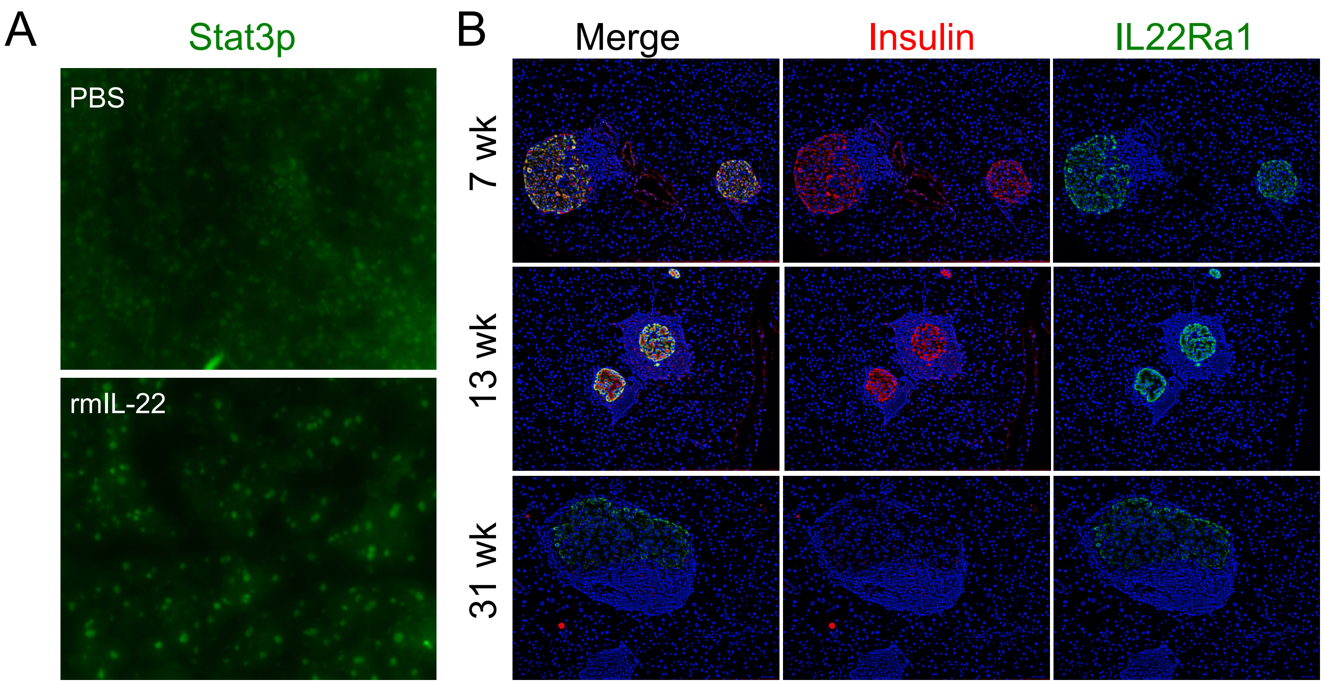

ESM Fig. 1: (**a**) Representative images of immunofluorescent staining with STAT3p antibody of NOD mice (n=4) pancreases treated with PBS or 200 ng/g rmIL-22 for 30 min. (**b**) Immunofluorescence was used to determine the levels of IL-22 receptor (IL-22RA1; green) in pancreatic islets (insulin; red) from NOD mice at 7, 13 and 31 weeks of age
